# NUV-Sensitive Silicon Photomultiplier Technologies Developed at Fondazione Bruno Kessler

**DOI:** 10.3390/s19020308

**Published:** 2019-01-14

**Authors:** Alberto Gola, Fabio Acerbi, Massimo Capasso, Marco Marcante, Alberto Mazzi, Giovanni Paternoster, Claudio Piemonte, Veronica Regazzoni, Nicola Zorzi

**Affiliations:** 1 Fondazione Bruno Kessler, 38123 Trento, Italy; acerbi@fbk.eu (F.A.); capasso@fbk.eu (M.C.); marcante@fbk.eu (M.M.); mazzi@fbk.eu (A.M.); paternoster@fbk.eu (G.P.); regazzoni@fbk.eu (V.R.); zorzi@fbk.eu (N.Z.); 2 Trento Institute for Fundamental Physics and Applications, 38123 Trento, Italy; 3 Department of Physics, University of Trento; 38123 Trento, Italy; 4 Broadcom Inc., 93049 Regensburg, Germany; claudio.piemonte@broadcom.com

**Keywords:** silicon photomultiplier (SiPM) technology, scintillation light readout, PET, Cherenkov light detection, cryogenic SiPM, liquid–Argon TPC, liquid, noble-gases scintillators, VUV-light detection, SiPM performance

## Abstract

Different applications require different customizations of silicon photomultiplier (SiPM) technology. We present a review on the latest SiPM technologies developed at Fondazione Bruno Kessler (FBK, Trento), characterized by a peak detection efficiency in the near-UV and customized according to the needs of different applications. Original near-UV sensitive, high-density SiPMs (NUV-HD), optimized for Positron Emission Tomography (PET) application, feature peak photon detection efficiency (PDE) of 63% at 420 nm with a 35 um cell size and a dark count rate (DCR) of 100 kHz/mm^2^. Correlated noise probability is around 25% at a PDE of 50% at 420 nm. It provides a coincidence resolving time (CRT) of 100 ps FWHM (full width at half maximum) in the detection of 511 keV photons, when used for the readout of LYSO(Ce) scintillator (Cerium-doped lutetium-yttrium oxyorthosilicate) and down to 75 ps FWHM with LSO(Ce:Ca) scintillator (Cerium and Calcium-doped lutetium oxyorthosilicate). Starting from this technology, we developed three variants, optimized according to different sets of specifications. NUV-HD–LowCT features a 60% reduction of direct crosstalk probability, for applications such as Cherenkov telescope array (CTA). NUV-HD–Cryo was optimized for cryogenic operation and for large photosensitive areas. The reference application, in this case, is the readout of liquid, noble-gases scintillators, such as liquid Argon. Measurements at 77 K showed a remarkably low value of the DCR of a few mHz/mm^2^. Finally, vacuum-UV (VUV)-HD features an increased sensitivity to VUV light, aiming at direct detection of photons below 200 nm. PDE in excess of 20% at 175 nm was measured in liquid Xenon. In the paper, we discuss the specifications on the SiPM related to different types of applications, the SiPM design challenges and process optimizations, and the results from the experimental characterization of the different, NUV-sensitive technologies developed at FBK.

## 1. Introduction

Silicon photomultipliers (SiPMs) are arrays of many (hundreds to tens of thousands) single-photon avalanche diodes (SPADs), each one with its integrated passive-quenching resistor, all connected in parallel to common anode and cathode. Like single SPADs, each SiPM cell works in Geiger mode and the output current (as well as amplitude or charge in an integration window) is the sum of all the cells, giving a signal proportional to the number of detected photons.

SiPMs have garnered growing attention as an alternative to the traditional photomultiplier tube in the detection of low photon fluxes thanks to a number of advantages typical of solid-state detectors and they are emerging as a very promising solution in many applications. In this scenario, it must be considered that different applications require different optimizations and improvements of silicon photomultiplier (SiPM) technology. In some cases, the changes to the original technology are so significant that a new SiPM technology is generated, serving a specific application or a class of applications. 

Traditionally, SiPMs have been used in the readout of the scintillation light, typically from lutetium-yttrium oxyorthosilicate (LYSO) crystals, for Time-of-Flight PET (ToF-PET). To achieve the best timing performance in this application, one of the most important figures of merit of the detector is photon detection efficiency (PDE); an example is shown in Reference [1]. Indeed, over the past few years, we have witnessed an extraordinary improvement of SiPMs in this regard. SiPMs from different manufacturers now feature peak PDE around 420 nm approaching 60% [2,3,4,5], while the PDE of the initial devices, available approximately 10 years ago, was in the order of 10–15% [6]. Additionally, the dark count rate (DCR) was reduced significantly and it currently ranges between 50 kHz/mm^2^ and 200 kHz/mm^2^. The reduction of DCR helps to improve timing performance because it limits baseline fluctuations before time pick-off of the signal generated by the 511 keV gamma photons used in PET [7].

On the other hand, thanks to the progress in SiPM technology, in recent years, there has been a growing interest in using these devices for many other applications. Among them, several big scientific experiments are considering SiPMs for the readout of liquid noble-gases scintillators, such as liquid Xenon (LXe) and liquid argon (LAr), as a replacement for the more conventional photomultiplier tubes [8,9]. In such applications, SiPMs must be operated successfully and with good performance at cryogenic temperatures, which constitutes a technological challenge for both the detector and its package. Moreover, very large sensitive areas are often required, setting strict constraints on the maximum DCR. Considering that LAr and LXe light emission peaks at 128 nm and 178 nm, respectively, in some experiments, high sensitivity in the vacuum-UV (VUV) is also needed to avoid using wavelength shifters [9].

A different type of experiment that will use SiPMs is the Cherenkov telescope array (CTA), in which the detectors are used to observe the Cherenkov light emitted in air showers initiated by high-energy to very-high-energy gamma-rays from both galactic and extragalactic sources [10]. In this case, SiPMs are operated in the presence of a significant amount of light generated by the night sky background and minimization of crosstalk probability is important to both reduce the rate of random triggers and to improve energy resolution. Similar requirements are set for the photodetectors by the second generation Extreme Universe Space Observatory Super-Pressure Balloon (EUSO-SPB2) [11], in which SiPMs are used to observe air showers produced by cosmic radiation in the night sky and face a significant amount of background light generated by the Earth.

In this work, we describe different SiPM technologies developed at Fondazione Bruno Kessler (FBK) and customized for different applications, outlining the most important design challenges. We start with the currently proven NUV-HD SiPM technology (near-UV sensitive, HD stands for “high-density” of cells in the SiPM), which was originally developed for ToF-PET application and features peak PDE in excess of 60% at 420 nm. Then, we go through the changes that are needed to obtain a good performance at cryogenic temperatures and constitute the new NUV-HD–Cryo technology. Finally, we describe modifications to the NUV-HD technology made to achieve better performance in the VUV range, obtaining the VUV-HD technology.

## 2. NUV-HD SiPM Technology

**Figure 1:**
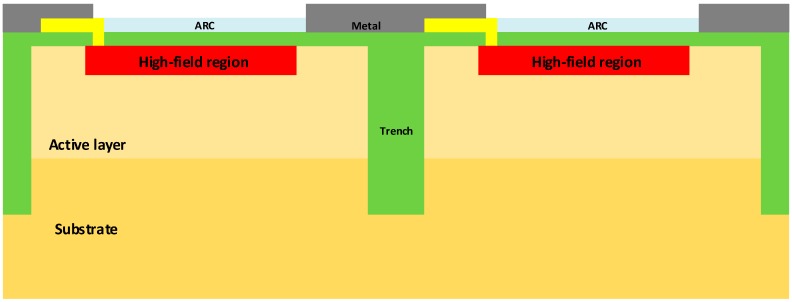
Structure of the cells of the NUV-HD technology (near-UV sensitive, HD stands for “high-density” of cells in the SiPM).

NUV-HD SiPM technology was introduced in 2016 and is described in Reference [2]. As shown in Figure 1, microcells are separated by deep trenches, which provide electrical isolation. Trenches are filled with silicon dioxide and, because of the different refractive index of silicon, they also provide partial optical isolation between microcells. The active area is defined by a high energy ion implantation, called deep implant or DI, which increases the electric field between the surface and the implant, to reach the avalanche condition. The space between DI and trench creates the so-called virtual guard ring (VGR), which prevents edge breakdown [12]. In this region, the cell is not sensitive to light. Typical breakdown voltage, V_BD_, is around 26.5 V.

The distance from DI to trench and the trench width are critical dimensions (CDs) of the technology and were minimized as much as possible, to increase the fill factor (FF) of the device, defined as:

(1) $$\mathrm{FF}=\frac{\mathrm{Active}\;\mathrm{Area}}{\mathrm{Cell}\;\mathrm{Area}}.$$

**Figure 2:**
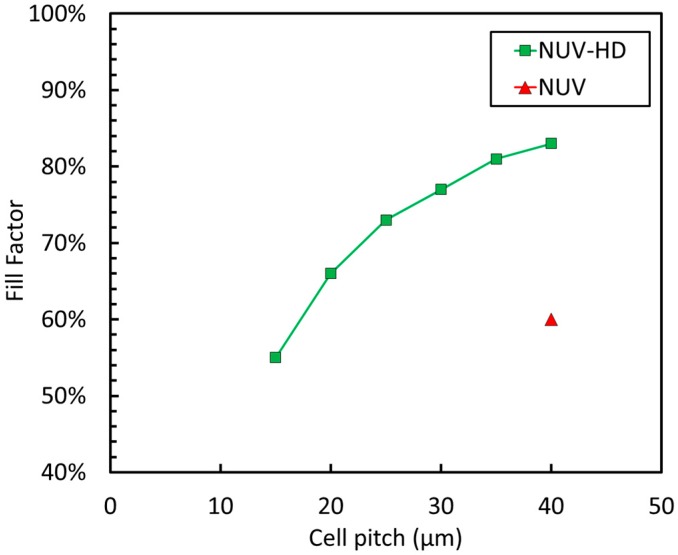
Fill factor (FF) as a function of the cell size for the NUV (red triangle) and NUV-HD (green squares) technologies.

The active area is the area of the microcell that is sensitive to light, corresponding, as a first approximation, to the area of the DI. Dead border at the edge of each microcell, including half trench width, is lower than 2 µm. Several cell sizes have been fabricated in the NUV-HD technology, each one featuring a different trade-off between PDE, correlated noise, and linearity. Indeed, linearity is strongly affected by cell density and cell recovery time [13]. It is interesting to observe the plot of the FF as a function of the cell pitch (CP) reported in Figure 2.

In Figure 2, we compare the recent NUV-HD technology to the older NUV technology (without trenches). We observe that all the cells built in a given technology lie on the same line, highlighted with the fit, which is determined by its critical dimensions. This can be considered the equivalent of a technology node for SiPM technologies. We also point out the improvement over the previous-generation NUV technology, which did not use trenches to isolate microcells and featured a dead border larger than 3.5 µm [14].

### 2.1. NUV-HD: PDE and Noise

**Figure 3:**
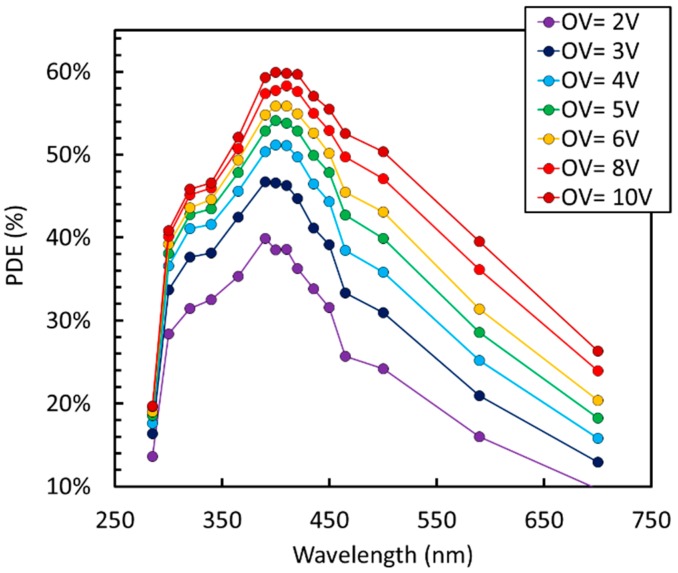
Photon detection efficiency (PDE) as a function of wavelength at different overvoltage (OV) for the NUV-HD 40 µm cell.

PDE vs. wavelength measured with the largest cell size is reported in Figure 3, for different values of the overvoltage (OV), i.e., the bias voltage above the breakdown voltage, V_BD_.

**Figure 4:**
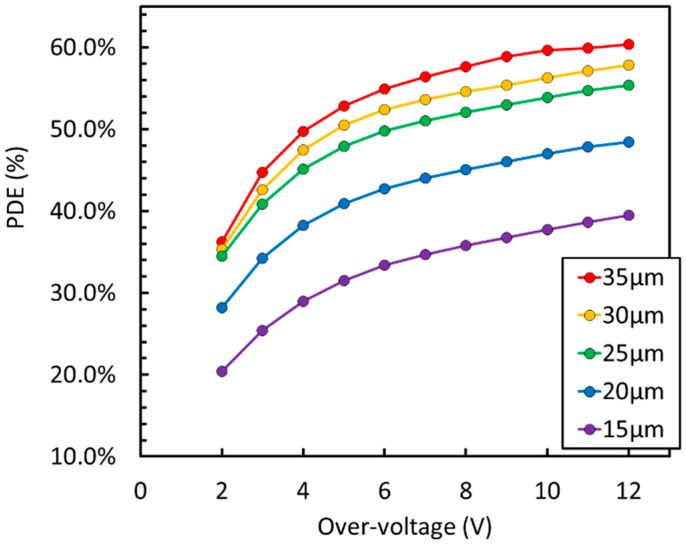
PDE at 420 nm as a function of the OV measured on different cell sizes of the NUV-HD silicon photomultiplier (SiPM) technology.

Peak PDE of 60% was measured at 410 nm, following the procedure described in Reference [15]. The detector was covered with protective silicone resin, transparent down to 300 nm. The curve matches LSO / LYSO emission (peaked around 420 nm) very well [16] and, together with very high peak efficiency, provides state-of-the-art timing performance [17] in the readout of scintillation light for ToF-PET applications, corresponding to a coincidence resolving time, or CRT, equal or lower than 100 ps FWHM (Full Width at Half Maximum). We also observed that PDE is close to 50% down to 300 nm, making this technology well suited for Cherenkov light detection. Figure 4 compares PDE measured at 420 nm on different cell sizes of the NUV-HD technology, showing the effect of the different values of FF.

**Figure 5:**
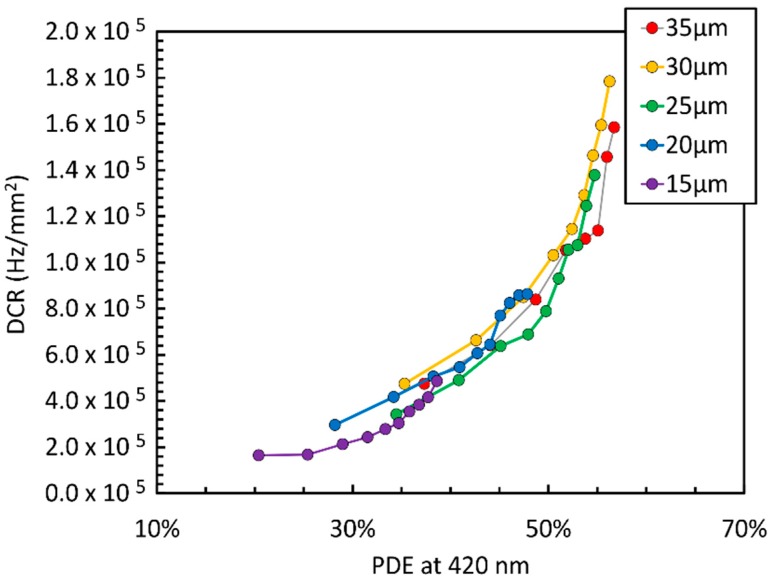
Dark count rate (DCR) as a function of the PDE at 420 nm for the different cell sizes of the NUV-HD SiPMs (from 15 µm to 35 µm).

The dark count rate of different NUV-HD cell sizes is reported in Figure 5.

It is important to outline that we plot the noise parameter as a function of the sensitivity at the wavelength of interest, in our case, 420 nm for LYSO readout. We believe that this is the most effective way of comparing different cell sizes of the same technology or even different SiPM technologies. The other typical way of plotting SPADs and SiPMs parameter is with respect to the overvoltage. In this case, smaller cells would feature lower DCR at any given OV, but that would happen mainly because of their lower FF, reflected also in a lower PDE. Thus, from the application point of view, when comparing the possible signal-to-noise ratios (SNR), such a plot would not inform correctly about the quality of the SiPM technology, which is what FBK, as a SiPM manufacturer, is mostly interested in. By contrast, in Figure 5, we observe that smaller cells feature a DCR comparable to that of larger cells, at the same level of PDE. We also point out that the DCR values shown in the figure are significantly improved compared to the ones reported in the first paper on NUV-HD technology [2].

**Figure 6:**
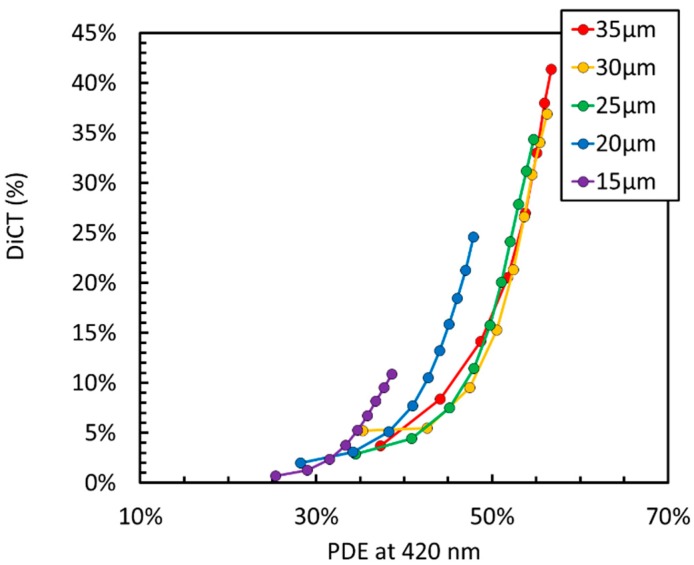
Direct optical Crosstalk (DiCT) as a function of PDE for the different cell sizes of the NUV-HD SiPMs (from 15 µm to 35 µm).

Another important noise source in SiPMs is correlated noise, constituting of optical crosstalk and afterpulsing. Figure 6 shows a plot of direct optical crosstalk (DiCT) vs. PDE at 420 nm for different NUV-HD cell sizes. DiCT is defined as in Reference [18].

In this case, we observed that bigger cells provide lower DiCT at the same level of PDE than smaller cells. Similarly to the DCR, this result is rather counterintuitive. Indeed, if DiCT were plotted as a function of OV, larger cells would feature significantly larger correlated noise because of the larger gain, i.e., because they produce a large number of secondary photons [2].

**Figure 7:**
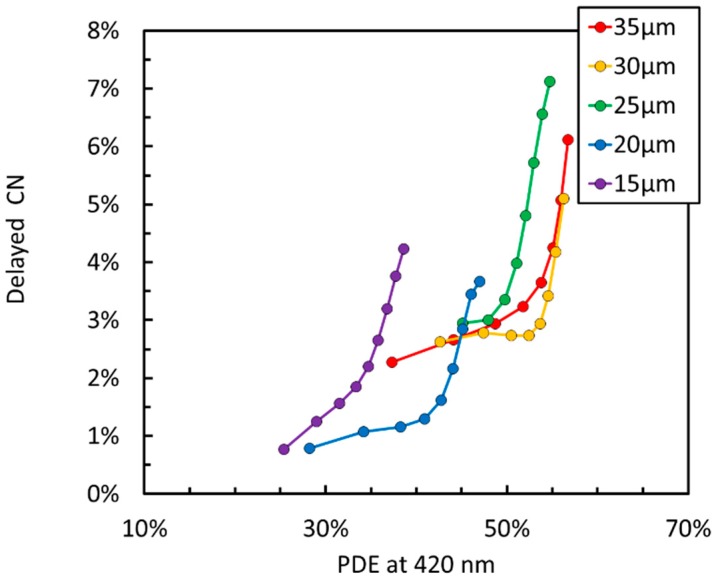
Delayed corrected noise (DeCN) as a function of PDE for the different cell sizes of the NUV-HD SiPMs (15 µm to 35 µm).

Figure 7 shows a plot of delayed correlated noise (DeCN) vs. PDE measured at 420 nm. We define DeCN as the sum of afterpulsing and delayed crosstalk, as defined in Reference [18]. Thanks to the technology improvement, described in Reference [19] for the NUV SiPMs and deep trench isolation employed in NUV-HD, the value is very low for all cell sizes, at the lower limit of the measurement sensitivity.

Another possible way to compare noise figures of different SiPMs is reported in Reference [20]. In this case, both SiPM PDE and noise are plotted as a function of the relative overvoltage, or OV_rel_, which is defined as the overvoltage divided by the breakdown voltage. The underlying assumptions are: (i) The PDE of the detector is determined, at first approximation, by the relative variation, E_rel_, of the peak electric field, E_p_, with respect to the peak electric field at breakdown, E_BD_; (ii) E_rel_ is proportional to OV_rel_.

This approach can also be effective for some comparisons and, under the assumptions reported above, theoretically correct. However, it cannot be applied to all types of SiPMs. In particular, it does not fit SiPM technologies fabricated at FBK well, because depletion at V_BD_ and above V_BD_ are significantly different and, thus, the second assumption is no longer valid. For FBK SiPM technologies, PDE increases slower with OV_rel_, because V_BD_ is kept low by the electric field design and this is done on purpose, to achieve improved V_BD_ uniformity and reduced V_BD_ temperature coefficient.

**Figure 8:**
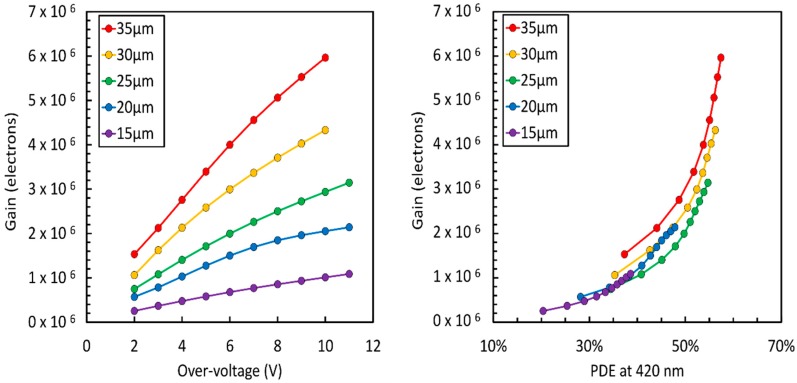
**Left**: Gain vs. overvoltage for all cell sizes. **Right**: Gain vs. PDE.

Figure 8, left, shows a plot of the gain as a function of the overvoltage for the different NUV-HD cell sizes. We define the gain as the average number of carriers passing through the high-field region of one cell when a photon or a dark count is detected, and no correlated noise event takes place [18]. As expected, gain increases with cell size, being proportional to cell capacitance. We also observe that, because depletion at V_BD_ and above V_BD_ are significantly different in NUV-HD technology, gain is less than linearly proportional to the overvoltage. From the measured gain values, we calculated a microcell capacitance, C_T_, ranging from 18 fF to 107 fF, for the 15 um and 35 um cells, respectively, at 6 V of OV. On the other hand, Figure 8, right, shows a plot of Gain vs. PDE measured at 420 nm. In this plot, differences between cell sizes are reduced because, although smaller cells feature a lower capacitance, they also need a higher value of OV to reach the same PDE as larger cells.

**Figure 9:**
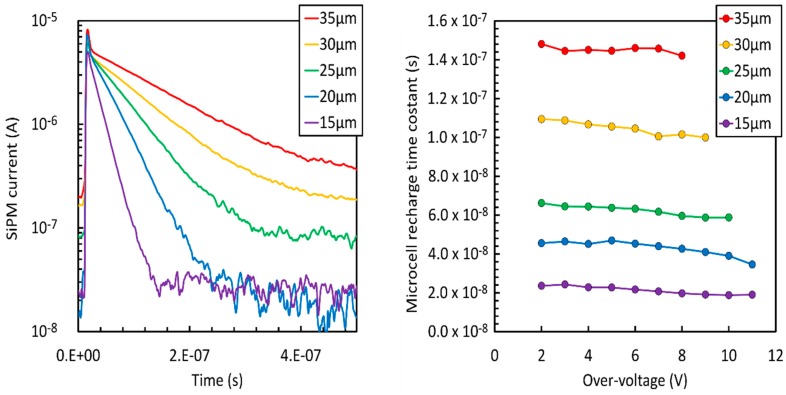
**Left**: Signal shape, in current, measured for all the cell sizes at 6 V overvoltage. **Right**: Recharge time constant, τ_r_, vs. overvoltage for all cells.

Figure 9, left, shows the current signals generated by 1 × 1 mm^2^ SiPMs with different cell sizes operated at 6 V overvoltage, when an avalanche was triggered in a single cell. The curves were measured using a fast, transimpedance amplifier, similar to the one reported in Reference [7]. The almost exponential trailing edge of the pulses is generated by the passive recharge of the microcells [21] and is independent on the amplifier response and on the detector active area, which, by contrast, mainly affects the rising edge of signals. In the plots, it is also possible to observe a fast peak on the rising edge generated by the capacitive coupling between anode of microcells and SiPM metal grid.

From these measurements, we calculated the recharge time constants of the microcells, τ_r_, which are plotted in Figure 9, right, as a function of the overvoltage. Because of the change of depletion below and above V_BD_, τ_r_ is reduced with increasing overvoltage. We also note that, for the same reason, the recharge transient, reported in Figure 9, left, is not purely exponential. The value of the quenching resistor, R_q_, in the microcells of the different samples was between 1.2 MOhm and 1.4 MOhm. While the plot of τ_r_ is useful to compare different cell sizes, we point out that the value of R_q_ is a relatively free design parameter, limited mainly by the need to achieve quenching at high values of OV and by the divergence of correlated noise, as described in the following section. Thus, it is possible to tune the value of R_q_ and obtain significantly different values of τ_r_, also shorter ones, according to different application needs.

### 2.2. Operational Limit of SiPM—Divergence of Correlated Noise

In many applications, the correlated noise of SiPMs should be reduced as much as possible because it increases the excess noise factor (ENF) of the photodetection system. This worsens the energy resolution in charge measurements or the photon number resolution in photon counting, as described in References [22,23,24]. The reduction of correlated noise, on the other hand, is also important in applications that do not require very high energy resolution. Indeed, we can define the number of correlated noise events generated by a single, primary avalanche as the excess charge factor (ECF). ECF can be expressed using the geometric series approximation:

(2) ECF≈1(1−PCN)

When the probability of having a correlated noise event, P_CN_, approaches one, ECF diverges [25]. The overvoltage at which this condition is met, OV_max_, can be considered the upper limit for the SiPM bias, although in most practical cases, SiPMs cannot be operated above P_CN_ = 0.5. We also point out that, for simplification, we have grouped all the correlated noise probabilities in a single number, P_CN_.

**Figure 10:**
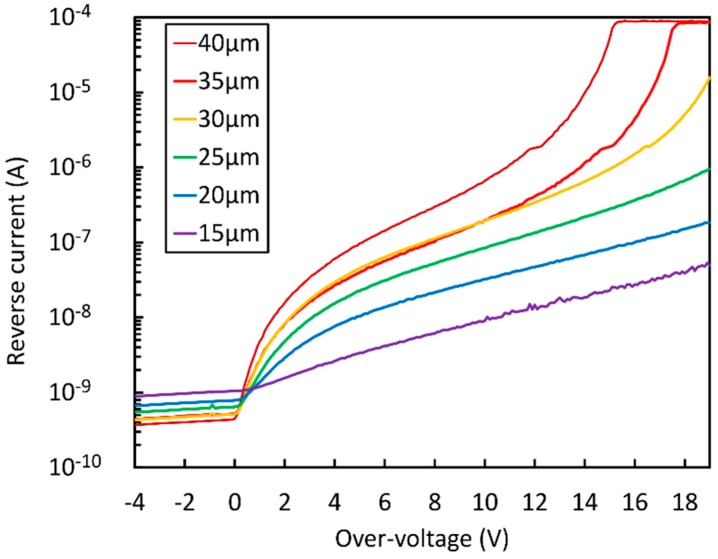
Reverse current as a function of the overvoltage for the different cell sizes of the NUV-HD SiPMs (from 15 µm to 40 µm).

Figure 10 shows the reverse current-voltage characteristics (IVs) measured on samples with different cell sizes of the NUV-HD technology. When OV_max_ is approached, the reverse IV rapidly diverges until it is limited by the instrument or by series resistances always present in the setup. This behavior can be called “second divergence” of the SiPM reverse IV curve, after the first one caused by the breakdown. We observe that OV_max_ is higher for smaller cell sizes, because they feature lower gain and, thus, lower correlated noise at the same overvoltage.

### 2.3. NUV-HD: Timing Performance

**Figure 11:**
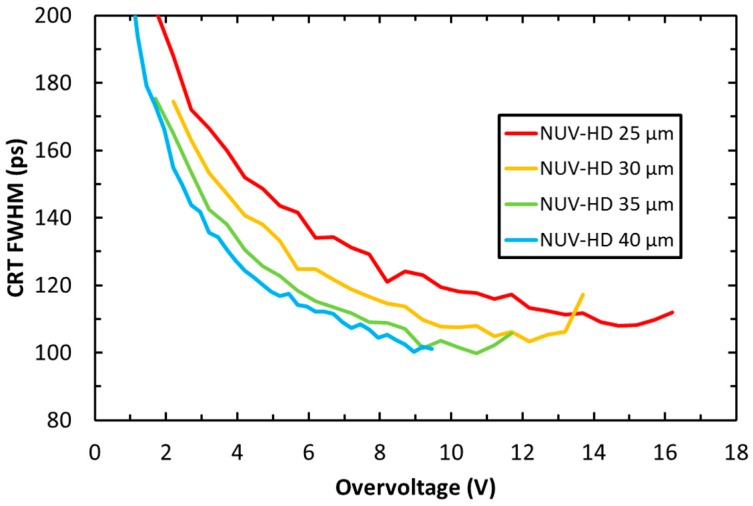
Coincidence reducing time (CRT) as a function of the OV, measured on different cell sizes of the NUV-HD technology.

As mentioned above, timing performance in scintillation light readout is mainly affected by SiPM PDE. However, it is also an important example of an application in which ultimate performance is also limited by the correlated noise, even though ENF is not the most important figure of merit. Figure 11 shows the coincidence resolving time (CRT) measured with NUV-HD SiPMs, featuring different cell sizes, coupled to LYSO scintillator crystals [26].

In the measurements, two 4 × 4 mm^2^ NUV-HD SiPMs were coupled to 3 × 3 × 5 mm^3^ LYSO(Ce) crystals for the detection of 511 keV gamma-photon emitted in coincidence by a ^22^Na source. We observe that, at low OV, CRT improves because SiPM Gain and PDE both increase with increasing OV, thus improving SNR with respect to electronic noise and photon time of arrival statistics, respectively. The effect of photon time of arrival statistics is described, for example, in Reference [1]. At any given value of OV, the performance of larger cell sizes is better because of a higher PDE and gain. However, the curve related to the 40 µm cell stops at 9 V because of the rapid divergence of the correlated noise, thus preventing further PDE and CRT improvements. A similar behavior is also observed in the other cell sizes, but, as expected, OV_max_ is shifted to higher values, effectively reducing the performance difference between different cell sizes. Although at the same OV smaller cells provide significantly lower performance, they can be biased at higher OV, partially compensating for the PDE difference, and thus also the CRT difference. We also note that, from a system point-of-view, smaller cell sizes provide reduced variations of CRT with respect to bias (CRT remains the same with small bias variations), although at the expense of a slightly worse optimal value.

An even better CRT of 75 ps FWHM was obtained using Calcium co-doped LSO, coupled to 40 μm-cell NUV-HD SiPMs [17]. Improved performance was obtained thanks to the faster light emission by the scintillator in the presence of Calcium co-doping. Considering a different scintillation material, NUV-HD SiPMs sensitivity to Cherenkov photons allowed a significant improvement of timing performance with a Bismuth germanate (BGO) scintillator. BGO features lower light yield (~8000 ph/MeV) and slower light emission (time constant ~300 ns), compared to LYSO [27]. On the other hand, BGO is also a Cherenkov radiator; thus, detection of a few prompt photons is expected to improve its timing performance. Indeed, beyond state-of-the-art CRT of 270 ps FWHM was measured in 2016, coupling 2 × 3 × 2 mm^3^ BGO crystals to 4 × 4 mm^2^ NUV-HD SiPMs, with a 40 μm cell size [28].

## 3. NUV-HD Low-Crosstalk

Certain applications require specifically that the probability of direct crosstalk, P_DiCT_, is minimized. As mentioned in the introduction, a typical example is CTA, in which SiPMs are used for the detection of the faint Cherenkov emission in the atmosphere caused by cosmic radiation, in the presence of a significant amount of night sky background, “NSB”, light. Since the Cherenkov signal has a low intensity, the PDE should be as high as possible in the spectral region of interest, which is estimated between 300 nm and 600 nm [29,30]. Similarly to many experiments, CTA requires reduction of correlated noise, including crosstalk and afterpulsing, to reduce ENF and, thus, improve energy resolution.

Reduction of the random events trigger rate induced by NSB is another important specification for CTA and is strongly dependent on P_DiCT_. Indeed, in the most common triggering scheme, SiPMs are self-triggered, with the trigger level, V_tr_, set at a certain threshold above the single photoelectron level. A higher CT probability increases the chances that a single photon from NSB generates a signal high enough to produce fake triggers. The rate of events caused by NSB depends on spectral filtering of photons impinging on the photo detector and on SiPM PDE, considering that NSB and Cherenkov signal feature different spectra. However, random events can also be strongly attenuated by increasing V_tr_ at the level of few photons, because the amplitude distribution of single, primary events in the presence of CT decreases almost exponentially with the amplitude. Different analytical models have been proposed for CT, such as, for example, in Reference [31]. On the other hand, since Cherenkov signal is faint, it is beneficial for sensitivity to reduce the triggering threshold as much as possible and to detect photons up to a wavelength of 600–650 nm. Because the NSB photon rate can be very high, the plot of P_DiCT_ vs. PDE can be considered one of the most important inputs to calculate the figures of merit for applications such as CTA.

In standard NUV-HD technology, microcells are separated by means of trenches filled with silicon oxide (SiO_2_). Although the main role of the trenches is to provide electrical isolation between adjacent cells, they are also effective for optical CT reduction. Even though the SiO_2_ is completely transparent to CT photons, an effective reduction of CT may be obtained thanks to the multiple reflection effect of photons through the Si/SiO_2_/Si interface. To further reduce crosstalk probability in NUV-HD technology, we redesigned the trench structure with the aim of reducing the total transmittance of CT photons through trenches. In this new design, we added a layer of highly doped polysilicon as light absorbing material inside trenches that separate microcells.

**Figure 12:**
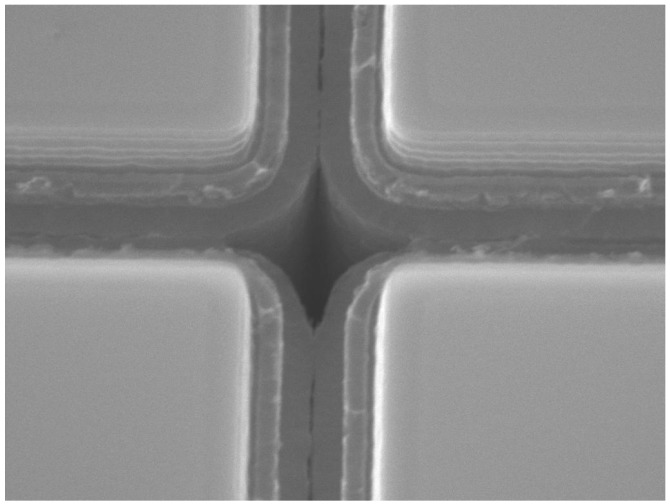
SEM image of SiPMs during the fabrication process, after the trench-filling step. Trenches are filled with SiO_2_ and highly doped polysilicon.

**Figure 13:**
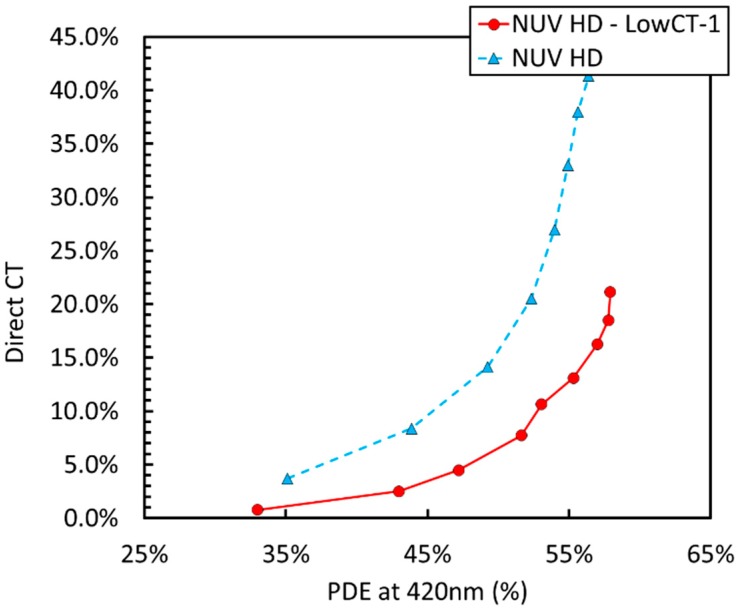
Probability of DiCT as a function of the PDE at 420 nm for standard NUV-HD and LowCT technologies.

Figure 12 shows an SEM image during the microfabrication process of a trench filled with the triple stack. The resulting structure is composed of a triple-layer stack SiO_2_/Poly-Si/SiO_2_. Such a structure is effective in reducing the CT mainly due to two factors: (i) The materials composing the stack show high-contrast refractive indexes, increasing the total reflection of light, which is back-scattered in the cell where it is originated; (ii) the polysilicon layer aids to absorb part of the light passing through the trench. Polysilicon reduces the transmission of photons generated during the avalanche, also called secondary photons, thus reducing the probability that they are detected by a neighboring cell. In this way, we obtained the LowCT-1 version of the NUV-HD technology: The technological trade-off is a minor increase of trench width, which causes a slight reduction of FF. Thus, a slightly higher overvoltage is needed to achieve the same PDE as standard NUV-HD technology. Figure 13 shows a plot of P_DiCT_ vs. PDE measured at 420 nm for the standard and LowCT-1 version of NUV-HD, measured on the 35 μm cell size.

We observed a reduction of the P_DiCT_ to approximately 35% of the value measured in standard technology, which implies a reduction of the transmission of CT photons between adjacent cells of the same magnitude.

**Figure 14:**
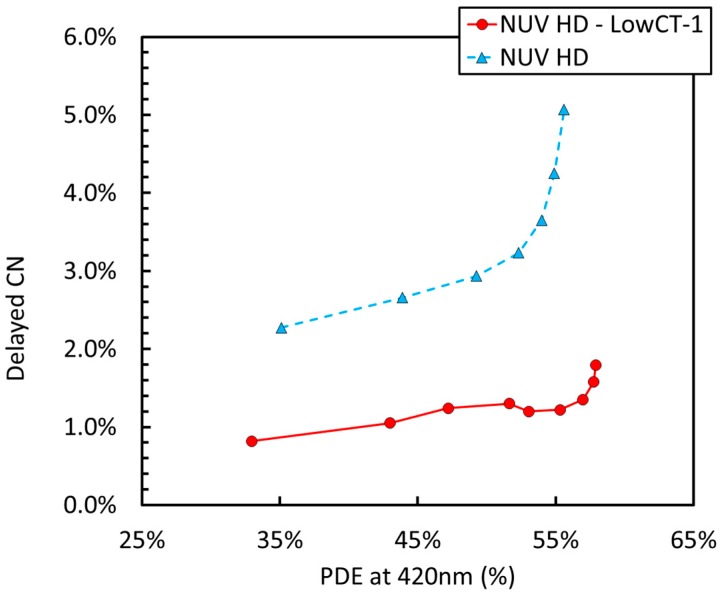
Probability of Delayed Correlated Noise (P_DeCN_) vs. PDE at 420 nm measured on NUV-HD and NUV-HD-LowCT-1 with 35 μm cell size.

We also noted that trenches are only a few micrometers deeper than the depleted part of the microcells, which is, as first approximation, the active region of the microcells. Thus, it is possible that secondary photons generate minority carriers in the undepleted silicon below the active region and that these carriers reach the active region by diffusion, generating a delayed event called delayed optical crosstalk, or DeCT [18]. As mentioned above, in order to reduce delayed crosstalk and, at the same time, part of the afterpulsing, FBK adopted a low-lifetime bulk, as described in Reference [19]. This solution if effective in reducing DeCT to an almost negligible level, as shown in Figure 14. This feature is common to all NUV and NUV-HD technologies developed or under development at FBK. Other technical solutions are possible to prevent minority carrier diffusion from undepleted regions below microcells, such as, for example, the use of a double epitaxy [12].

Although the introduction of polysilicon provides a significant reduction of CT, the attenuation of transmission of secondary photons between microcells is not complete. Other manufacturers have developed a different process, introducing metal in trenches for a total reflection of secondary photons at microcell borders [32]. In this configuration, CT is further attenuated but not completely removed, because there are still different paths for the secondary photons to reach neighboring cells. Indeed:

*1.*  Secondary photons can exit the SiPM surface and be guided to other cells by SiPM packaging, for example, protective resin;
*2.*  DeCT may be not affected;
*3.*  It has also been hypothesized that secondary photons with longer wavelengths might travel through the bulk and be reflected from the backside, usually metal finish of the detector [24].

The efficiency of the last effect is uncertain, though, but it may be relevant in certain device configurations, such as, for example, when the wafer is thinned to a few tens of micrometers for the application of Through-Silicon Vias (TSVs).

**Figure 15:**
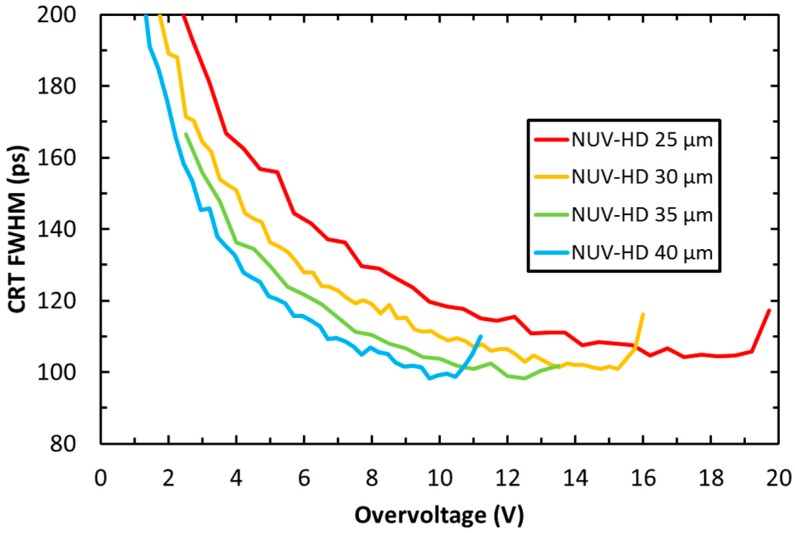
CRT as a function of the OV for NUV-HD LowCT SiPMs having different cell sizes.

On the other hand, we should consider that when a SiPM is employed in a system, the reflection of secondary photons by surrounding material becomes relevant. Thus, secondary photons exiting the SiPM surface can be reflected back and generate crosstalk in microcells far away from the initial avalanche. This effect is called external crosstalk, or ExtCT, and, depending on the system configuration, can have an intensity comparable to that of internal crosstalk, or IntCT [25]. A typical case is the readout of scintillation light for timing, described in Section 2.3. Indeed, scintillation wrapping is normally optimized to reflect photons, also including, as a side effect, avalanche secondary photons. In this case, the ultimate reduction of IntCT provides minor improvements in performance because total CT probability is determined by ExtCT. For example, Figure 15 shows a limited improvement in the timing performance of NUV-HD–LowCT-1 devices, compared to the one of NUV-HD devices, reported in Figure 11, notwithstanding the 50% lower CT probability.

## 4. NUV-HD–Cryo SiPM Technology

Experiments employing SiPMs for the readout of liquid scintillators usually require that the detectors are operated at cryogenic temperatures and cover very large sensitive areas. For example, the ongoing upgrade of the DarkSide experiment, called DarkSide-20k (DS20k), will use approximately 15 m^2^ of SiPMs for the double-sided readout of a dual-phase Time Projection Chamber (TPC), filled with LAr [8]. Coating with TetraPhenylButadiene (TPB) will shift LAr light from 128 nm to 420 nm, to better fit SiPM responsivity.

Because of the large photosensitive area in DS20k, to reduce complexity and power dissipation of the readout electronics, an unusually large SiPM area is connected to a single channel of the readout electronics, in the order of 25 cm^2^. This can be implemented either by connecting several dies in parallel or using more sophisticated techniques, such as series or series-parallel connections [33,34]. Limiting power dissipation to a few thousands of readout channels is very important in a cryogenically cooled environment. On the other hand, the use of SiPMs for the readout of very large photosensitive areas also descends from the fact that they aim at replacing photomultiplier tubes, which were used in previous generation experiments with up to an 8′′ diameter [35].

Since the SiPM area connected to a single front-end channel is very large, a reduction of detector noise is of paramount importance. This is especially true in experiments looking for rare events, such as DS20k, in which the rate of randoms per channel should be minimized. As a consequence, DS20k originally set a specification of a DCR of less 1 Hz/mm^2^ on the detector.

### 4.1. Reduction of DCR at Cryogenic Temperatures

To meet such stringent specifications, two NUV-sensitive technologies available at FBK were evaluated at cryogenic temperatures, the standard NUV-HD (standard field, “SF”) and the modified NUV-HD-LF (low field, “LF”) [36]. The two technologies are equivalent in every aspect except for the peak value of the electric field at breakdown, which is reduced in the LF version. This is obtained using a deeper high-energy n-type implant to define the high-field region in each microcell. As a consequence, V_BD_ is shifted to 32.5 V in NUV-HD-LF.

The first result of cryogenic operation of SiPMs is the reduction of the breakdown voltage. The effect happens because of an increased mean free path between scattering of carriers that drift in the high-field region. Thus, a lower electric field is needed to reach breakdown condition. V_BD_ changes from 26.5 to 21.4 for the SF technology and from 32.5 to 27 for the LF technology, when cooling from room temperature down to 77 K.

**Figure 16:**
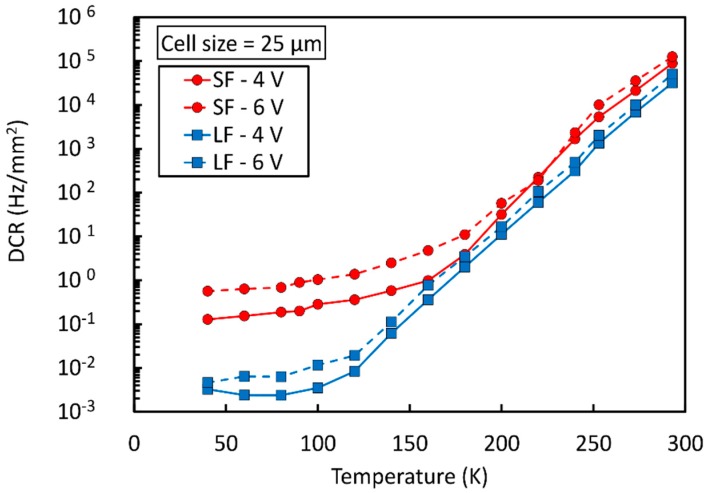
DCR as a function of temperature for the NUV-HD (SF) and NUV-HD-LF (LF) technologies at two values of OV. Measures taken from Reference [36]. SF: Standard field, LF: Low field.

Figure 16 shows the DCR vs. temperature measured on SiPMs with a 25 µm cell size for the two technologies at two different values of OV.

In the high-temperature part of the plot, carrier generation and, thus, DCR, is dominated by field-enhanced thermal generation, described by Shockley-Read-Hall statistics [37]. This is confirmed by an activation energy of 0.43 eV (SF) and 0.48 eV (LF), which can be extracted by the Arrhenius plot calculated from the data in Figure 16. We notice that the DCR of standard-field (SF) and low-field (LF) technologies are comparable near room temperature. The higher value of the activation energy in LF is explained by a lower field-enhanced effect.

Below a given temperature, reduction of noise reaches a plateau because it becomes dominated by direct, band-to-band tunneling, which is weakly dependent on temperature, through silicon bandgap variations only. We can observe that, in this regime, the LF reaches a DCR more than 20 times lower than the SF. Additionally, we notice that the difference between the DCR at different OV is larger in the SF, which is again a confirmation of a higher, field-dependent tunneling effect.

The result obtained with the NUV-HD-LF at low temperatures is remarkable. Below 80 K, we measured a DCR of a few mHz /mm^2^. To put things in perspective, we would need, on average, three days to observe a single dark count in a 25 µm cell. On a different scale, a 100 cm^2^ SiPM tile has a total DCR of less than 100 Hz. This level of DCR is enabling for the use of SiPM in the readout of liquid scintillators. It even compares favorably to the DCR that can be achieved by Photomultiplier Tubes (PMTs) at these temperatures, which is in the order of 1 kcps [38].

**Figure 17:**
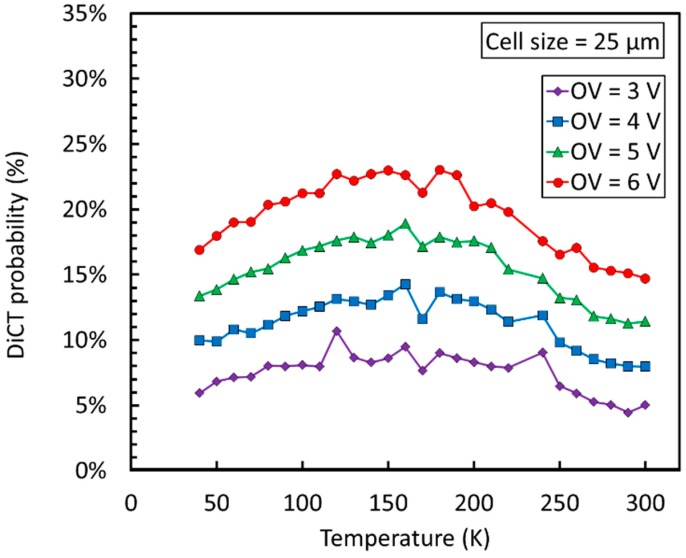
DiCT as a function of the temperature and overvoltage for NUV-HD-LF SiPMs. Measures taken from Reference [36].

Considering correlated noise, Figure 17 shows a plot of the direct crosstalk (DiCT) as a function of the temperature for the NUV-HD-LF technology with a 25 µm cell size.

We observed that DiCT is weakly dependent on temperature. Without further analysis, the most likely explanation is that emission of secondary photons by the avalanche does not undergo major changes with cooling. In this condition, the observed dependence of DiCT probability on temperature can be attributed to the changes in gain and PDE caused by the different depletion of the microcell at different temperatures at a given overvoltage, because V_BD_ is shifted. This hypothesis does not exclude a change in the emission of secondary photons with temperature, which should be verified by further, dedicated measurements.

### 4.2. Reduction of Afterpulsing Probability at Cryogenic Temperatures

**Figure 18:**
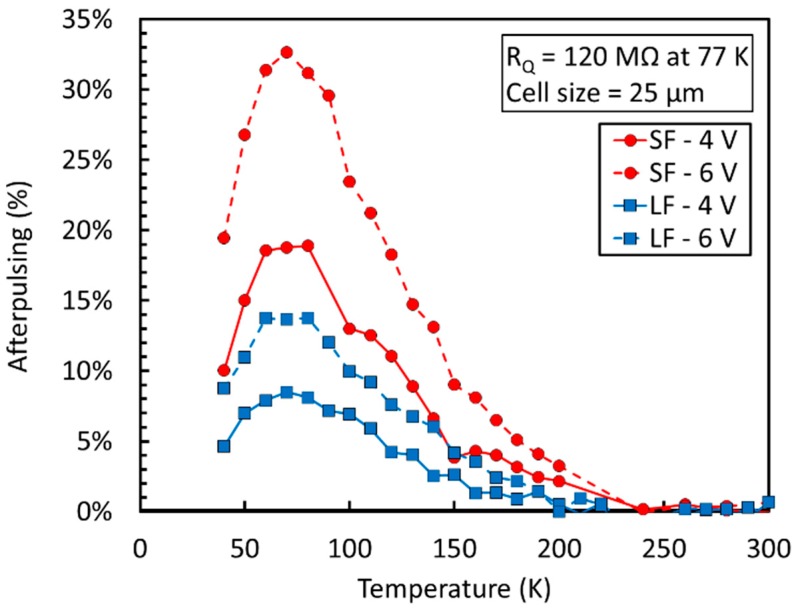
Afterpulsing probability (AP) as a function of the temperature for the NUV-HD and NUV-HD-LF technologies. Measures taken from Reference [36].

Compared to DiCT, we observed a radically different behavior of the afterpulsing probability (AP), which is plotted in Figure 18 for the SF and LF technologies, with a 25 µm cell size.

AP is very low near room temperature in both cases, close or below the sensitivity of the measurement technique. Below 150 K, however, it increases rapidly, reaching its maximum around 80 K. The increase can be attributed to a longer emission time constant at cryogenic temperatures of carriers captured by trapping centers. Our hypothesis is that, at room temperature, a significant fraction of carriers is emitted by trapping centers when the microcells are still partially discharged after the primary avalanche: Thus, carriers have a lower probability of triggering an afterpulse. However, the emission time constant is increased at cryogenic temperatures, thus increasing AP.

We point out that the value of the quenching resistor, which is built in polysilicon, also undergoes significant variations with temperature. Correspondingly, the microcell recharge time constant, τ_r_, for the NUV-HD-LF increases exponentially with cooling and changes from 80 ns at 293 K to 3.5 µs at 77 K. This effect is competing with the increase of the emission time constant and it allows to keep AP increase under control. We also noticed that LF is effective in reducing AP by 50% in similar conditions.

On the other hand, the AP increase reported in Figure 18 is undesirable for several reasons. First, the need to increase τ_r_ distributes the delivery of the signal charge produced by the SiPM over a long time period. We obtained a long single cell response (SCR) with reduced peak amplitude. Thus, for a given design and power consumption of the single channel of the front-end, the SNR gets worse both in the case of amplitude and charge measurements. Increasing SNR as much as possible is very important in the readout of very large area SiPMs, because the large capacitance at the input of the preamplifier increases the output electronic noise and reduces the peak signal amplitude by low-pass filtering [39]. The problem can be partially mitigated, especially in amplitude measurements, by the introduction of a fast capacitive coupling between anode of microcells and SiPM metal grid, by a properly sized C_q_, as described in Reference [39].

The second, more important problem caused by the AP increase is the reduction of OV_max_. Indeed, reducing its value limits the maximum PDE and gain of the SiPM, thus reducing the SNR for electronic noise, significantly affecting performance even without taking into account the effects of ENF. Moreover, the value of OV_max_ features a very strong dependence on even small variations of SiPM parameters, because it is caused by diverging phenomena. Thus, as will be shown in the next paragraphs, we expect a relatively large spread of OV_max_ in a given SiPM population. From the point of view of the experiment design, such a condition should be avoided, and a robust safety margin should be kept from OV_max_ for system reliability, further limiting the maximum bias applied to the SiPM and reducing detector performance.

**Figure 19:**
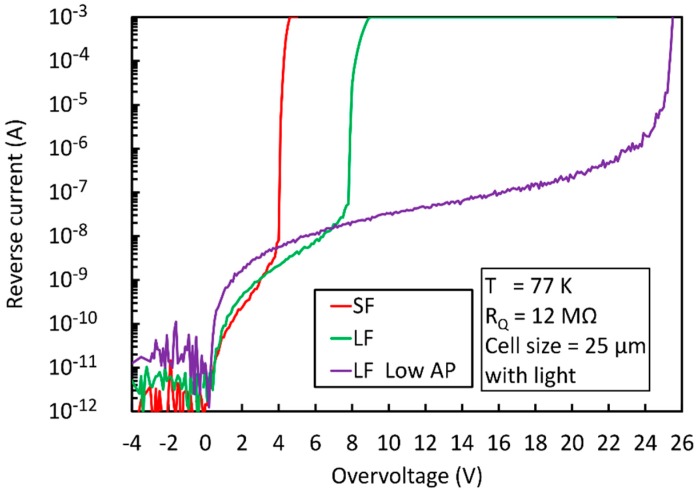
Reverse current-voltage characteristics measured at 77 K for the NUV-SF, NUV-LF and NUV-LF-LowAP SiPM.

To overcome these limitations, we identified a modified fabrication process strongly reducing the cryogenic AP of the SiPM. The NUV-HD-LF technology was then fabricated with the standard and LowAP process splits. Figure 19 shows the reverse IVs, measured at 77 K in the three cases.

Plotted curves were measured on SiPMs with 25 µm cells and a total active area of 25 mm^2^. To better highlight divergence of correlated noise, measurements were carried out under faint illumination, because dark current at 77 K was below the sensitivity of the employed instruments. The value of the quenching resistor, R_q_, at 77 K was reduced to 12 MOhm in all samples, to enhance the difference in afterpulsing probability at cryogenic temperatures. Correspondingly, the recharge time constant was 480 ns. This value is significantly lower than 3.5 μs, characteristic of the devices reported in Figure 18. We noticed that NUV-HD-LF features higher OV_max_ at 77 K than standard NUV-HD SiPMs, but the value is still much lower than the one obtained at room temperature, as shown in Figure 10. On the contrary, the LowAP split, plotted in purple, shows only a minor reduction of OV_max_ when compared to room temperature, recovering almost all the useful bias range.

**Figure 20:**
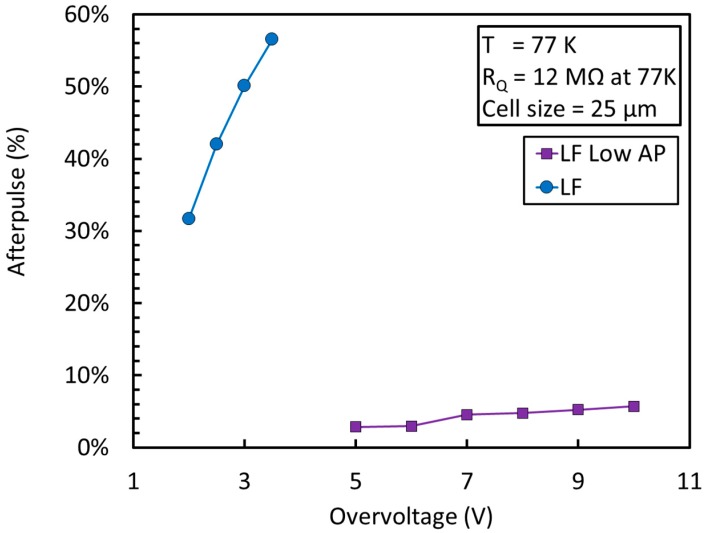
AP as a function of the OV at 77 K for the NUV-HD-LF and NUV-HD-LF-LowAP SiPMs.

Figure 20 shows a plot of the AP as a function of the OV measured on the standard and LowAP splits of NUV-HD-LF at 77 K. Also in this case, a value of 12 MOhm at 77 K for the quenching resistor was employed for both samples. We observed a reduction of AP of more than a factor of 10 in the same conditions. The result is very important, because it shows the possibility of operating the NUV-HD-LF technology at cryogenic temperature with low correlated noise and extended bias range and can be considered the second enabling modification of the NUV-HD technology for its use in the readout of liquid scintillators.

### 4.3. Stability of Quenching Resistor with Temperature

The reduction of AP allowed the use of a reduced value of the quenching resistor and, thus, of the microcell recharge time constant at cryogenic temperatures, with significant advantages for the readout electronics. On the other hand, the standard quenching resistor employed in NUV-HD technology is made of poly-Silicon and undergoes significant variations with temperature, changing by a factor of ~60 from 293 K to 77 K. A resistor with a value of ~6.5 MOhm at 77 K would have a value of 100 kOhm at room temperature, preventing correct SiPM operation above 2–3 V, because the avalanche would not be properly quenched [40].

**Figure 21:**
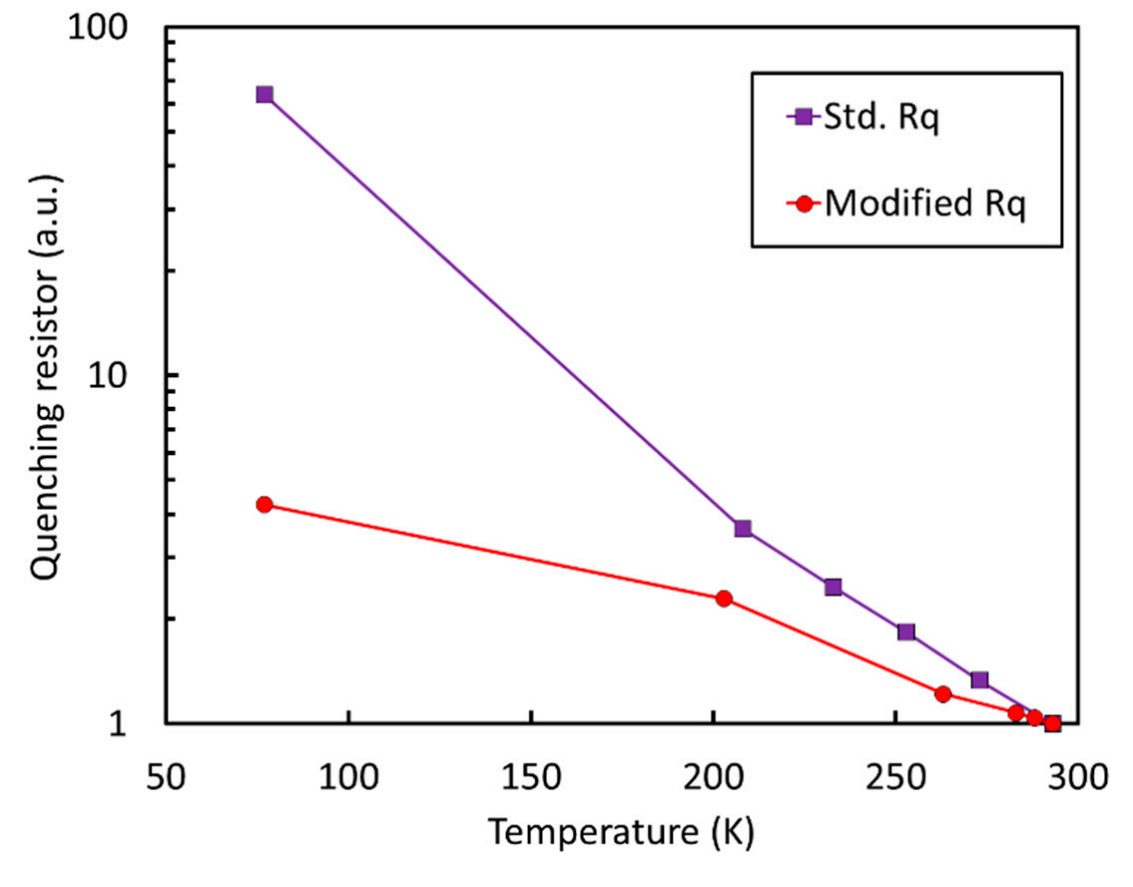
Variations of the quenching resistor value for the standard and modified quenching resistor used in NUV-HD-LF, LowAP SiPMs.

To allow a proper operation of SiPM both at room and cryogenic temperatures, we developed a technological variant of the polysilicon quenching resistor, with significantly reduced temperature coefficient. The new resistor was obtained using a significantly lower sheet resistance of the polysilicon combined with a different layout. Figure 21 compares the relative variation of the resistor values in the two cases.

The new type of resistor changes its value only by a factor of ~4 from room temperature to cryogenic temperatures. Correspondingly, the recharge time constant of the 25 µm microcells changes from 65 ns to 270 ns at 293 K and 77 K, respectively.

### 4.4. NUV-HD–Cryo: PDE and Noise

From the combination of the technology optimizations described in the previous sections, we obtained the NUV-HD–Cryo SiPM technology. It includes the following technology upgrades, aimed at a better performance in cryogenic applications:

*1.*  Low electric field inside the junction for reduced DCR;
*2.*  LowAP split for reduced afterpulsing probability;
*3.*  Modified quenching resistor with reduced temperature coefficient and further reduced value of 6.5 MOhm at 77 K.

Table 1 summarizes the characteristics of the NUV-HD–Cryo technology, compared to standard NUV-HD.

**Figure 22:**
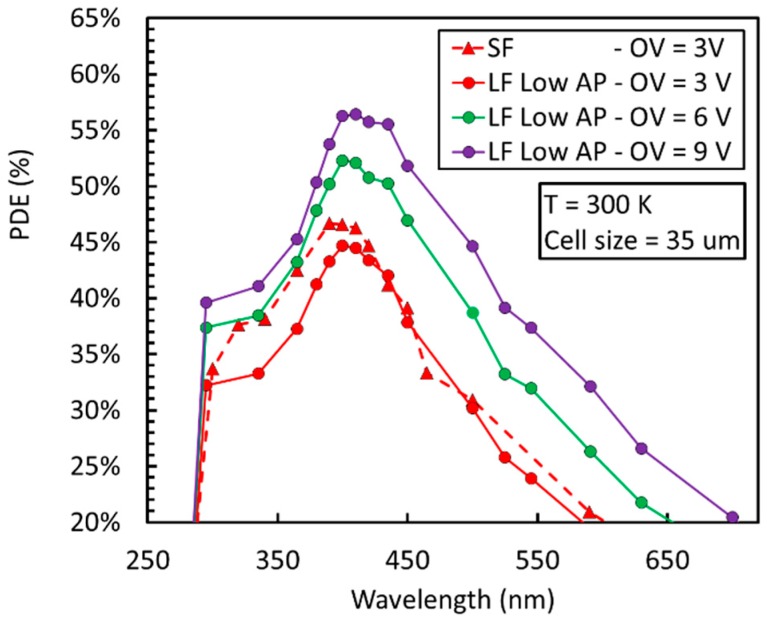
PDE measured at 293 K on 35 μm cell, NUV-HD–Cryo SiPM, compared to that of a standard NUV-HD SiPM, with the same cell size.

The PDE measured at room temperature on the NUV-HD–Cryo SiPM is shown in Figure 22 for the 35 µm cell, which features a higher FF than the 25 μm cells, reported in Table 1, and, thus, a slightly higher PDE. Measurement was carried out using protective silicon resin, applied on top of the SiPM entrance window.

**Figure 23:**
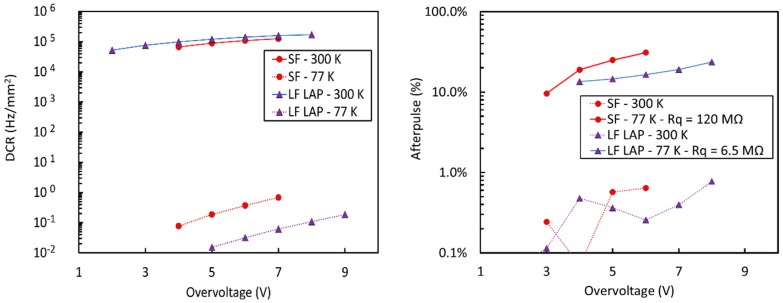
(**left**) DCR measured at 293 K and 77 K on 25 μm cell NUV-HD and NUV-HD–Cryo SiPMs; (**right**) Afterpulsing probability measured at 293 K and 77 K on 25 μm cell NUV-HD and NUV-HD–Cryo SiPMs with values of quenching resistor of 120 MOhm and 6.5 MOhm, respectively.

We noticed that the increase of PDE with respect to overvoltage is slower in NUV-HD–Cryo than in NUV-HD, because of the lower field in the high-field region. A setup for measurement of PDE at 77 K is not yet available at FBK, although preliminary measurements showed a reduced difference at 420 nm between values measured at 293 K and 77 K. Because of the absence of direct PDE measurements, noise figures of NUV-HD and NUV-HD–Cryo technologies are plotted as a function of OV in Figure 23. We observed that, even if the afterpulsing probabilities measured at 77 K are not very different in the two technologies, they are obtained with very different microcell recharge time constants (see Table 1).

## 5. VUV-HD SiPM Technology

Many big scientific experiments use wavelength shifters, such as TPB, for the detection of the scintillation light emitted by LAr and LXe. This is the case of DarkSide-20k and DUNE. On the other hand, direct detection of LXe scintillation at 178 nm and LAr scintillation at 125 nm, with a high detection efficiency, could provide significant advantages in terms of performance and system simplification. Two main factors limit the sensitivity of standard NUV-HD SiPMs in the VUV spectral range:

- Presence of antireflective coating (ARC) not optimized for VUV light detection. Typical ARC consists of a multilayer stack of SiO_2_ and Si_3_N_4_, which minimize light reflection in the visible spectral range. On the other hand, these materials, transparent to the visible light, start to absorb at wavelength <250 nm and <150 nm, for Si_3_N_4_ and SiO_2_, respectively, affecting the external Quantum Efficiency (QE) of the sensor.
- Low penetration depth of VUV photons in silicon. Photons in this spectral range are thus absorbed in the first few nanometers from the front surface, where the generated electron–hole pairs have a high probability to recombine at the highly defective front surface. This effect may strongly affect the collection probability of generated carriers and, thus, the internal QE.

In this context, FBK started the development of VUV-sensitive devices, aiming at extending PDE as much as possible towards shorter wavelengths. Developments started from the NUV-HD technology. Technical challenges were: (i) To improve external QE by tuning the ARC and removing the dielectric layers, on top of silicon, that absorbs VUV photons; (ii) to improve internal QE by carefully optimizing the undepleted implants that form the light entrance window.

In the first version of the VUV sensitive SiPMs (named VUV-HD), we mainly worked on ARC optimization, by preserving, at the same time, a good passivation quality of the front surface.

**Figure 24:**
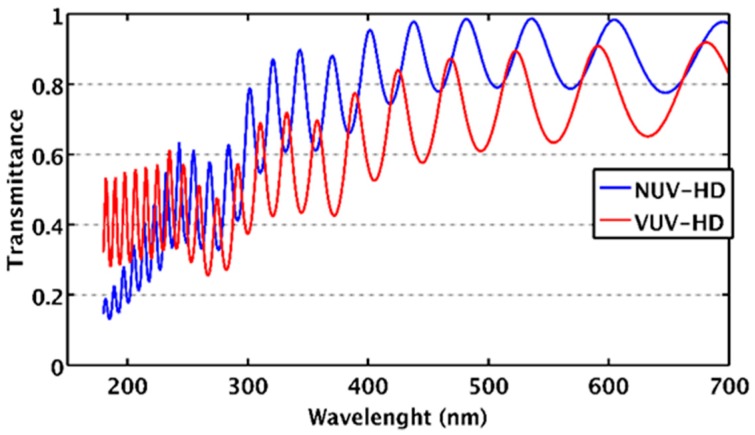
Calculated transmittance in silicon of normal incident light on the NUV-HD ARC (blue line) and on the modified ARC of VUV-HD SiPMs.

Figure 24 shows the total transmittance in silicon of VUV-HD ARC compared to standard NUV-HD. Optical simulations were carried out under the assumption of normal light incidence and by considering air as surrounding medium. It is worth noting that NUV-HD has a higher transmittance at wavelength >300 nm, but it rapidly decreases at shorter wavelengths, mainly due to Si_3_N_4_ absorption. On the other hand, the VUV-HD ARC transmittance remains in the range 30–50% at wavelength <200 nm. The oscillations in the spectrum are due to light interferences in the multilayer stack.

**Figure 25:**
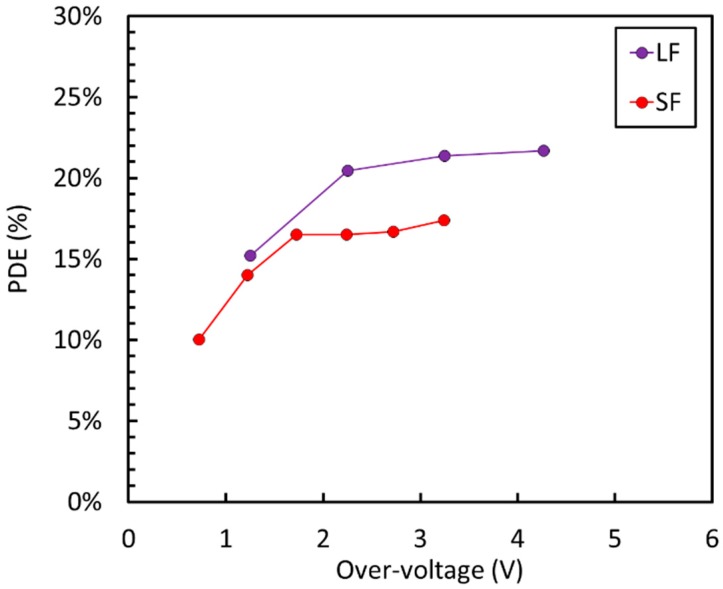
PDE at 175 nm, measured on VUV-HD SiPM with 35 μm cell size in LXe. Measures taken from Reference [41].

By applying these changes to the original technology, we obtained the VUV-HD technology. The first characterization of the devices was carried out in collaboration with the nEXO project [9]. Figure 25 shows the measured PDE vs. OV measured at 169 K in LXe at Stanford, for both low-field and standard-field technologies, as described in Reference [41].

We observed the high maximum PDE value around 22%. The upper limit of the bias applied to the SiPM was determined by the increase of correlated noise at low temperatures, described in previous sections. The main factor limiting PDE in this case seems to be the reflectivity of photons by the ARC, as could be inferred by the simulations reported in Figure 24, which shows a loss of about 50% of incident photons due to reflection losses. The high reflectivity of the SiPM surface at 178 nm is due to the large differences of the indices of refraction between silicon (nSi = 0.682), the silicon dioxide (nSiO_2_ = 1.61), and LXe (nLXe = 1.66).

R&D on VUV-HD technology is still ongoing. Main improvements planned in the near future are: (i) Application of the cryogenic SiPM technologies to the VUV-HD, to reduce DCR and AP; (ii) reduction of ARC reflectivity, to enhance the VUV PDE. In this regard, the principal limiting factor is that SiO_2_ is not the optimal choice to match the refractive indexes of LXe and silicon in the VUV. In future developments, we will explore ARC based on different materials (MgF, Al_2_O_3_) or multilayer stacks, which could be effective in reducing reflectance and thus enhance the external QE of SiPMs.

## 6. Conclusions

In the paper, we discussed the development and optimization of NUV-sensitive, FBK SiPM technologies and their customization according to different application needs. The original technology, developed in 2015, is the NUV-HD: It features a cell size ranging from 15 um to 40 um, cell separation by means of deep trench isolation, peak PDE in excess of 60% at 420 nm, and DCR below 100 kHz/mm^2^ at 50% peak PDE. The sensitivity spectrum matches LYSO light emission very well and, indeed, the first application of the technology was scintillation light readout for PET. In this case, the most important parameter is the maximum PDE that can be obtained before the divergence of correlated noise, when a scintillator is placed on top of the SIPM. State-of-the-art performance was measured in LYSO(Ce) and LSO(Ce,Ca) readout, obtaining a CRT of 100 ps FWHM and 75 ps FWHM, respectively.

NUV-HD technology also features a high sensitivity close to 50% down to 300 nm. This feature is ideal for Cherenkov light detection, both from scintillators to improve timing with prompt photons for PET and from air showers initiated in the atmosphere by cosmic radiation, as in CTA.

On the other hand, application as CTA requires a reduction of correlated noise and, in particular, of direct crosstalk. Taking into account these specifications, customized, NUV-HD-LowCT-1 technology was developed using light absorbing material to fill trenches. P(DiCT) was reduced from 20% to 6%, at 50% PDE at 420 nm.

A collection of three technological improvements was needed to build the NUV-HD–Cryo technology, optimized for low-temperature operation, down to 70 K. In this case, the driving application was the readout of scintillation light in large, double-phase TPC filled with LAr, in DarkSide-20k experiment. Peculiar features of NUV-HD–Cryo technology are: (i) Reduction of DCR at low-temperature; (ii) reduction of afterpulsing at low temperature; (iii) reduced variations of the value of quenching resistor with temperature. Remarkably low DCR of less than 5 mHz/mm^2^ was measured at 77 K with this technology. In this condition of operation, a 100 cm^2^ SiPM tile has a total DCR of 100 Hz.

Finally, thanks to optimized antireflective coatings and entrance window, a PDE in excess of 20% was measured at 175 nm on VUV-HD SiPMs operated at 169 K. A target application of this technology is the direct detection of scintillation light produced by an LXe scintillator.

The described improvements in detector technology are very interesting from a scientific perspective and outline a recent trend in SiPM R&D. Increase of PDE of SiPMs is approaching saturation due to physical limits to FF increase, external and internal QE. Similarly, a decrease of DCR is starting to be limited to values determined by purity of available starting materials and field-enhanced effects. Thus, it difficult to expect big year-to-year improvements in major SiPM parameters, as was common in early development phase.

On the other hand, it is becoming evident that a single SiPM technology cannot fulfill the needs of very diverse applications. Customized technologies provide a significant performance increase in specific use cases and applications. One example of such technologies, not reported in this work, is FBK UHD-SiPMs with an ultra-high density of cells, up to 50 k cells/mm^2^, for an extended linear range and increased radiation hardness [42]. Another example is the development of NIR-sensitive SiPMs, which are very different from NUV-sensitive technologies and, as a first approximation, require n-on-p junction, increased epitaxial layer thickness and, possibly, light trapping techniques. Thus, while standard SiPM technology is slowly reaching maturity, recent trends favor technology customization.

## Figures and Tables

**Table 1:** Comparison of the main features of NUV-HD and NUV-HD–Cryo technologies, 25 μm cell size.

	NUV-HD		NUV-HD–Cryo	
	293 K	77 K	293 K	77 K
Breakdown Voltage (V_BD_)	26.5 V	21.5 V	32.8 V	27.1 V
V_BD_ temperature coefficient	27 mV/°C	20 mV/°C	35 mV/°C	21 mV/°C
DCR (5 V)	100 kHz/mm^2^	0.2 Hz/mm^2^	100 kHz/mm^2^	2 mHz/mm^2^
Quenching resistor	1.9 MΩ	120 MΩ	1.6 MΩ	6.5 MΩ
CT probability (5 V)	20%	16%	9%	13%
AP probability (5 V)	<1%	25%	<1%	12%
OV_max_	12 V	8 V	25 V	20 V
Recharge time constant	80 ns	3.5 µs	65 ns	270 ns
Peak PDE (5 V, 410 nm)	48%	-	37%	-
